# Protective effect of Mumiju against acetic acid-induced ulcerative colitis in rats

**Published:** 2018

**Authors:** Nadar Shahrokhi, Zakieh Keshavarzi, Mohammad Khaksari Haddad, Fereshteh Amirafzali, Shahriar Dabiri, Nava Shahrokhi

**Affiliations:** 1 * Physiology Research Center, Institute of Neuropharmacology, Kerman University of Medical Sciences, Kerman, Iran*; 2 *Natural Products and Medicinal Plants Research Center, North Khorasan University of Medical Sciences, Bojnurd, Iran*; 3 *Department of physiology and pharmacology, North Khorasan University of Medical Sciences, Bojnurd, Iran*; 4 * Endocrinology and Metabolism Research Center, Institute of Basic and clinical physiology, Kerman University of Medical Sciences, Kerman, Iran*; 5 * Payame Noor University, Isfahan, Iran*; 6 *Department of Pathology, University of Medical Sciences, Kerman, Iran*; 7 *School of Medicine, Kerman University of Medical Sciences, Kerman, Iran*

**Keywords:** SOD, GSH, MDA, Mumiju, Rats, Colitis

## Abstract

**Objective:**

In this study, we elucidated the ameliorative effect of aqueous extract of leaves of Mumiju against acetic acid-induced experimental colitis in male rats.

**Materials and Methods:**

The animals were randomly divided into four groups (n=7) including I: control group, II: vehicle group (injected with 2 ml acetic acid (4%) intra rectally), III and IV: treatment groups which received Mumiju (250 mg/kg) orally or intraperitoneally for 4 consecutive days after ulcer induction. Ulcer index, severity of inflammation, colonic levels of superoxide dismutase (SOD), glutathione (GSH), and malondialdehyde (MDA), and histological changes were recorded after the treatment regimen of 4 days.

**Results:**

The ulcer index, severity of inflammation and colonic MDA levels were increased following intrarectal instillation of acetic acid. Also, acetic acid significantly decreased the SOD and GSH levels. Treatment with Mumiju for 4 days exhibited significantly lowered oxidative stress, while elevated of SOD and GSH levels. Regenerative-healing patterns also was seen by histopathological findings after treatment with Mumiju.

**Conclusion:**

The present investigation demonstrates that Mumiju could be regarded as a herb with potent therapeutic value in the amelioration of experimental colitis in laboratory animals by modulation of oxidant- antioxidant system.

## Introduction

Ulcerative colitis and Crohn’s disease are chronic, relapsing, immune-mediated disorders that are collectively referred to as inflammatory bowel diseases (IBD). Etiology and pathogenesis of IBD remain unclear, although environmental factors, along with genetic factors, are suggested to be involved in IBD pathogenesis (Fiocchi, 1998 ▶; Loftus, 2004 ▶). Prolonged or insufficient activation of the intestinal immune system contributes to the pathological events of chronic mucosal inflammation (Sartor, 1997 ▶). A growing number of scientific papers suggest that inflammatory bowel diseases originate from an abnormal immune response to normal bacterial flora. Deregulated immune system activation results in the overproduction of reactive metabolites of oxygen and nitrogen that will induce intestinal and colonic injuries and dysfunctions observed in IBD. In many studies, it has been shown that antioxidants can ameliorate the ulcerative colitis (Nosalova et al., 2000 ▶). 

A number of chemical drugs are available for treatment of peptic ulcer, but some side effects and drug interactions make them difficult to use. Thus, development of new anti-ulcer drugs, and search for novel molecules have been extended to herbal drugs that would offer better protection and can decrease the relapse rates (Ghannadi et al., 2011 ▶; Ghosal et al., 1988 ▶). Iran has a unique climatic condition that contributes to the growth of a wide range of medicinal plants (Goel et al., 1990 ▶). 

Mumijo, also known as Shilajit, Salajit, Shilajatu, Mumi, or Mummiyo originates from the snow petrels, *Pagodroma nivea*. It is a pale brown to blackish-brown exudation of variable consistency extracted from layers of rocks in many mountain ranges of the world (Agarwal, 2007 ▶). Mumiju is a famous traditional medicinal herb used for the treatment of different diseases. Mumiju has shown beneficial effects for treatment of gastrointestinal disorders (Shakurov, 1965 ▶; Kel’ginbaev, 1973 ▶), bone pains and fractures (Mirza, 2010 ▶; Garedewa, 2004 ▶). The Asian Mumiju contains 20% minerals, 15% protein, 5% lipids, 5% steroids and also some carbohydrates, alkaloids and amino acids (Aiello, 2011 ▶; Ghosal, 1993 ▶). Also, this substance has exhibited memory improving, neuroprotective, anti-inflammatory and anti-oxidant effects (Spassov, 1994 ▶; Bhattacharya, 1995 ▶). It is assumed that biological effect of Mumiju is due to the presence of di-benzo-alpha-pyrone, humic acid and folic acid substances (Agarwal, 2007 ▶; Bhattacharya, 1992 ▶). 

Until now, the probable modulatory role of Mumiju in colon inflammation has not been declared; hence, we designed the current examination to assess the possible modifying effect (s) of Mumiju extract on acetic acid-induced ulcerative colitis in male rats.

## Materials and Methods

**Animals**

In this study, male Albino N-Mary rats, with a mean weight of 180-250 g, were under constant environmental conditions with equilight and dark cycles and had free access to a proper diet chow and water ad libitum. All animals were handled according to the guidelines approved by the Animal Care and Use Committee of Faculty of medicine, Kerman University of Medical Sciences, Kerman, Iran.

**Preparation of the extract**

Mumiju was prepared from the local residents of Sardoiyeh in Jiroft, Kerman, Iran. After at least 2-3 times washing, it was dried, powdered and dissolved in normal saline (to obtain a concentration equal to that being used by local residents). Then, it was placed on shaker for 24 hr, centrifuged (at 5000 g for 10 min) and sterilized in an autoclave. The prepared powder was finally dissolved in normal saline in order to be injected at the dose of 250 mg/kg/day. All the test samples were administered by oral gavage or intraperitoneally in a volume of 2 ml, once a day to each rat (Phaechamud et al., 2008 ▶; Ghaaazi et al., 2018 ▶).

**Induction of colonic inflammation in rats**

All animals were fasted overnight, with access to water ad libitum, before induction of colitis and were anesthetized by ether inhalation. A polypropylene tube with 2mm diameter was inserted through the rectum into the colon to a distance of 8 cm. A solution of two ml acetic acid (Merck, Germany): saline (4%, v/v) was instilled. The rats were then maintained in a Trendelenburg position for 30 sec to prevent early leakage of the intracolonic instillation (Keshavarzi et al., 2014 ▶).

**Animal grouping**

Adult male rats were randomly divided into five groups (7 rats in each) as follows: I: control group without induction of colitis (intact group); II: vehicle group in which rats received equal-volume of Mumiju vehicle (normal saline 2 ml/kg, orally) after ulcer induction and III and IV: treatment groups that received Mumiju (250 mg/kg) orally (gavage) or intraperitoneally (IP) in a volume of 2 ml for 4 consecutive days after ulcer induction. On the 4th day after colitis induction, all these treatments were given for four days by using oral gavage or intraperitoneally (Shahrokhi et al., 2015 ▶).

**Assessment of colon macroscopic damage**

A segment of the colon, 8 cm in length and 3 cm proximal to the anus was excised, opened longitudinally and washed in saline buffer. These tissue specimens were weighed. A pathologist who was unaware of treatment conditions recorded macroscopic and histological damages. The criteria for macroscopic evaluation relied on a previously validated scoring system (0-4) (Morris et al, 1989). The scores were: 0=no ulcer; 1=mucosal erythema only; 2=mild mucosal edema, slight bleeding or slight erosion; 3=moderate edema, bleeding ulcers or erosions; and 4=severe ulceration, erosions, edema and tissue necrosis. Ulcer area was measured using 3M® scaled surgical transparent tape, which was fixed to a light and transparent sheet. Each cell on the tape was 1 mm^2^ in area and the number of cells covering the ulcerated area of each specimen was counted. Ulcer index was measured by summing the ulcer score and the ulcer area of each tissue specimen. Ulcer index was calculated according to the following formula: UI=UN+US+UA×10^-1^, where UI=ulcer index, UN=ulcer number, US=ulcer score, and UA=ulcer area.

**Assessment of colon histological damage**

For histological examination, colon tissues were separately fixed in 10% formalin, dehydrated, paraffin embedded, processed, sectioned as 4 µm-thick sections, and stained with haematoxylin and eosin (HE). Inflammation severity (0=none, 1=slight, 2=moderate, and 3=severe) was assessed in HE-stained, coded sections using a validated scoring scheme described by Cooper et al (Cooper et al., 1993) and Dieleman et al (Dieleman et al., 1998) with some modifications. The stained sections of colon were examined for any inflammatory changes like infiltration of the cells, necrotic foci and damage to tissue structures like payers patches, damage to nucleus. Crypitis, crypt-abscess, goblet cells depletion and also inflammatory cells infiltrates in the lamina propria and colonic wall were estimated.

**Biochemical studies**

The rest of the colon was used for the assessment of superoxide dismutase (SOD), glutathione peroxidase (GPX) and lipid peroxidation (malonaldehyde (MDA)) levels. The samples (n=7) were homogenized in 10% (w/v) of ice-cold potassium phosphate buffer (pH 7.4) using Elvenjan homogenizer (Remi Motors Ltd., Mumbai). Lipid peroxidation (LPO) was evaluated by measuring MDA using thiobarbituric acid method described by Ohkawa et al. (Ohkawa et al, 1979), and expressed as nmol/mg protein. GPX and SOD content were also measured using the Randox assay kits, and the content of GPX and SOD were given as U/µg protein. Estimation of protein content follows the method of Lowry et al. (Lowry et al., 1951). 

**Statistical analysis**

The values were expressed as mean±S.E.M. The statistical analysis was carried out by one way analysis of variance (ANOVA) followed by multiple comparison test of Tukey–Kramer. P values <0.05 were considered significant.

## Results

**Biochemical studies**

**Figure 1:**
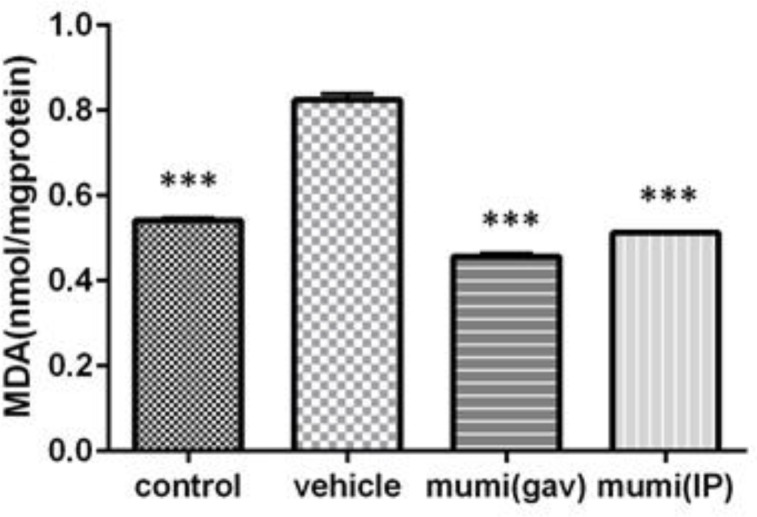
MDA levels at different groups. Data are presented as mean±SEM. ***: p<0.001, vehicle group vs other groups.

**Figure 2:**
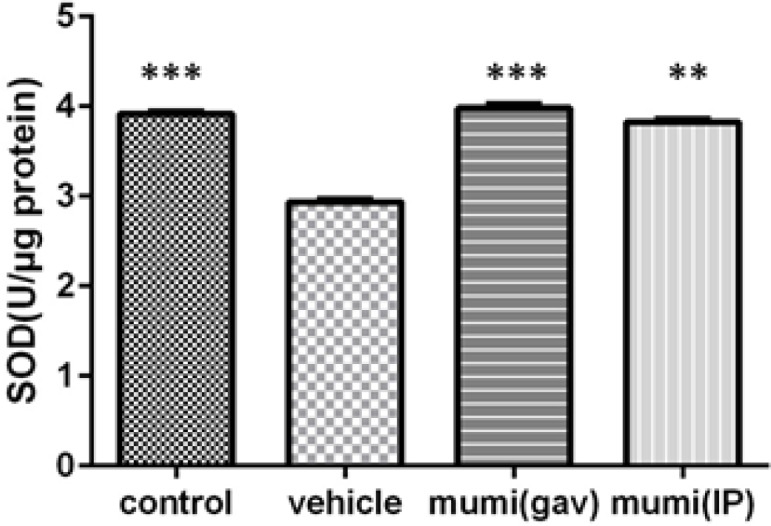
SOD levels (U/µg protein) in different groups. Data are presented as mean±SEM. ***: p<0.001, vehicle group vs other groups.

**Figure 3:**
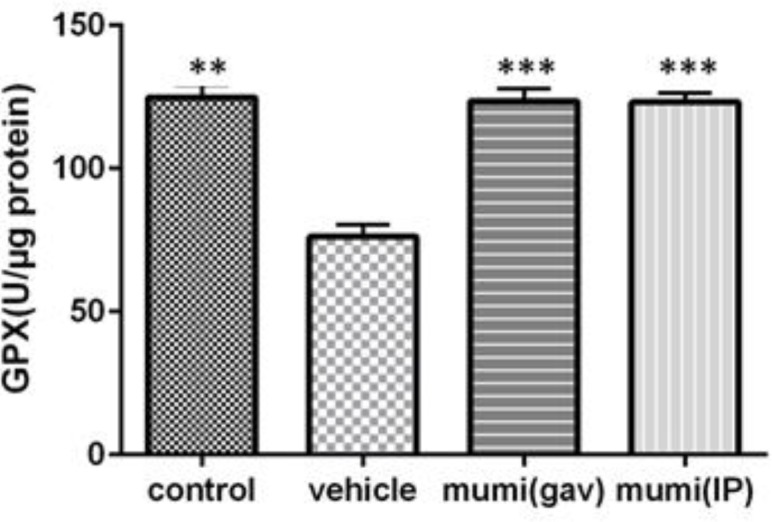
GPX levels (U/µg protein) at different groups. Data are presented as mean±SEM. ***: P< 0.001, **: P<0.01, vehicle group vs other groups.

Intra-rectal administration of acetic acid significantly increased concentrations of MDA (0.82±0.01 nmol/mg, p<0.001), while decreased SOD (2.93±0.04 u/µg, p<0.001) and GPX levels (76.23±0.41 u/µg, p<0.001) in colonic tissue compared to normal control rats. In terms of SOD and GPX activities, Mumiju-treated groups showed significant increases in comparison to the vehicle group (p<0.001). In Mumiju groups, MDA levels were significantly lower than those of the vehicle group (p<0.001). Treatment with Mumiju at 250 mg/kg significantly (p<0.001) reduced the alterations in these biochemical parameters and restored them to the normal levels (Figure. 1-3).

**Ulcer index**

**Figure 4:**
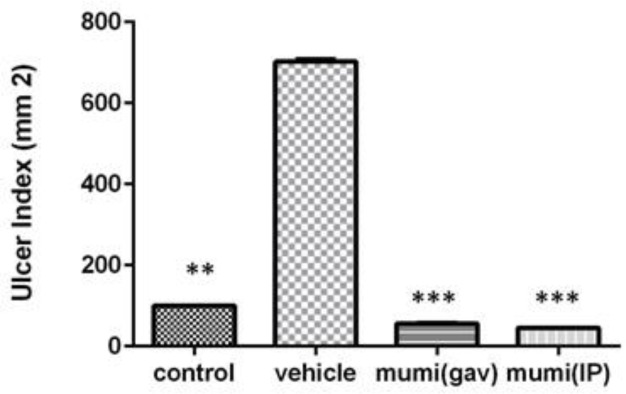
Index ulcer (mm^2^) in different groups. Data are presented as mean±SEM. ***: p<0.001, vehicle group vs other groups.

The mean ulcer index of vehicle group was 702±8.66 mm^2^ which showed high ulcerogenic effect of acetic acid. Treatment with Mumiju (250 mg/kg) for 4 days, decreased the ulcer index following administration via both routes (56.19±2.88 mm^2^ and 45.84±1.62 mm^2 ^for Mumiju-gavage and Mumiju-IP, respectively) (p<0.001) as compared to vehicle group (Figure 4).

**Inflammation severity**

**Figure 5:**
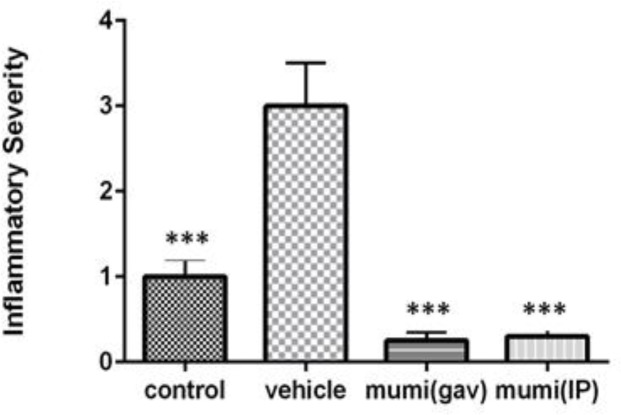
Inflammatory severity in different groups. Data are presented as mean±SEM. ***: p<0.001, vehicle group vs other groups.

 Acetic acid induced a vigorous inflammatory response (3±0.3) in vehicle group. Treatment with Mumiju (250 mg/ kg) for 4 days, decreased this inflammation following administration via both routes (0.25±0.1 and 0.3±0.1 for Mumiju-gavage and Mumiju-IP, respectively) (p<0.01 and p<0.05, respectively) as compared to vehicle group (Figure 5).

**Figure 6:**
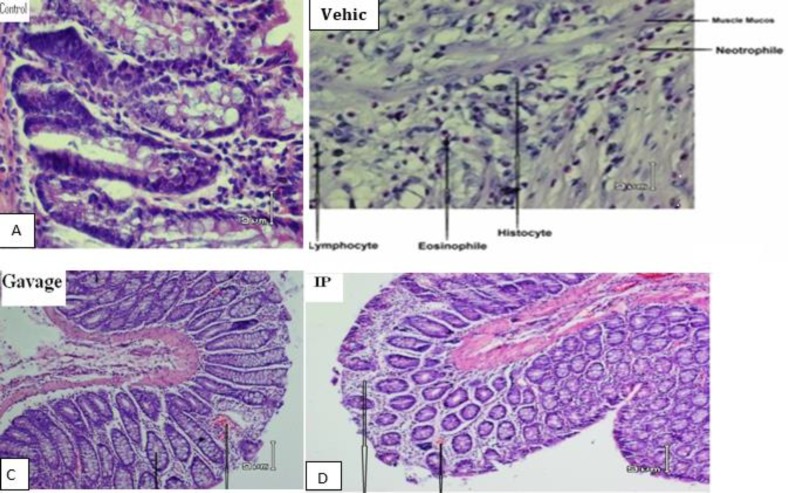
Photomicrographs showing histopathological changes in different groups. (A) The normal colon morphology of the control animals (H&E; original magniﬁcation, ×20). (B) The histopathoglogical changes in the acetic acid-induced colitis in the vehicle group. These changes include gastric ulcer with transmural necrosis (H&E; original magniﬁcation, ×10). (C, D) The histology changes in Mumiju group including extensive repair tissue, treatment withMumiju extract (250 mg/kg) (oral gavage) or (intraperitoneally) attenuated the extent and severity of cell damage (H&E; original magniﬁcation, ×10).

**Histopathological studies**

Acetic acid induced extensive destruction of epithelium (ulceration), submucosal edema, areas of hemorrhage, infiltration of inflammatory cells in the lamina propria, cryptitis, crypt-abscess and goblet cell depletion of the colonic glands. Mumiju (250 mg/kg) showed remarkable recovery of colonic mucosa from acetic acid-induced colitis damage (Figure 6).

## Discussion

Inﬂammatory mediators such as reactive oxygen species, eicosanoids and vasoactive amines play a prominent role in acetic acid-induced colitis and some other inflammatory models (Carty et al., 2000 ▶; Keshavarzi et al., 2012 ▶). The principle mechanisms involved include destruction of colon structure and mucosa barrier by chemical injury, enhanced vessel permeability, inﬂammatory mediator levels, and ﬁbrin hydrolysis, and disturbance of cruor process.

In the present study, we declared that Mumiju administered via both routes significantly inhibited the activity of lipid peroxides in the colonic tissue. It is therefore reasonable to assume that treatment with Mumiju improves colonic oxidative balance in animals with colitis, because Mumiju was able to lessen the level of MDA, a good indicator of lipid peroxidation. Increased lipid peroxidation products in colonic tissue can start a vicious cycle that creates more reactive metabolites, which in turn deplete the cellular antioxidants such as vitamin C and E and finally worsen inflammation and ulceration (Carty et al., 2000 ▶). In agreement with the current findings, we previously showed that Mumijo have antiulcer activity via reduction of gastric acid secretion and pepsin levels in an acetic acid-induced gastric ulcer model (Shahrokhi et al., 2015 ▶). 

Our study also showed that treatment with Mumiju increased the GPX levels and restored its levels near the normal levels. GPX is involved in different mechanisms including the synthesis and repair of DNA, recycling of vitamins C and E, prevention of free radicals-induced damage, improvement of the antioxidant activity of vitamin C, and facilitation of the transport of amino acids and plays a principle role in detoxification (Chavan et al., 2005 ▶). 

The protective effect of Mumiju on acetic acid-induced colitis in rats was well confimed by the histopathological studies. In agreement with our study, the incidence of ulcers induced by cysteamine in rats and histamine in guinea pigs also significantly reduced in duodenal ulcers following pretreatment with Mumijo (Goel et al., 1990 ▶). The antioxidant and anti-inflammatory effects of this exudation have been also extensively presented in some other studies. For instance, it was shown that alterations in the antioxidant status following ulceration, implies that free radicals may be associated with gastric mucosal damage in rats (Repetto et al., 2002 ▶). The results obtained from Mumiju-treated acetic acid-induced colitis in the present study, is in well correlation with some previous studies. Ghazi et al., showed Shilajit treatment reduced IL-10, IL-6, IL-1β, and TNF-α levels, following hepatic injury induced by administration of a single dose of acetaminophen 500 mg/kg (Ghaaazi et al., 2018 ▶). In addition, Mumijo had significant anti-inflammatory effects in chronic inflammation. There are some documents showing that Mumijo increases superoxide dismutase, catalase, and glutathione peroxidase activities in rats (Bhattacharya et al., 1995 ▶). Also, Mumijo can significantly decrease carrageenan-induced edema in rats paw (Ghosal et al., 1990). In some scientific reports, it has also been described that Mumijo has anti-allergic effects on histamine release and causes mast cells degranulation (Ghosal et al., 1989 ▶).

Mumijo, as an ancient therapy for treatment of various diseases, gained attention because of its anti-oxidant, immune- modulating, and anti-aging effects. It contains humus (60–80 %), benzoic acid, hippuric acid, fatty acids, ich-thyol, albuminoids, dibenzo-a-pyrones, essential oils, and various vitamins and minerals, such as B1 and B2 (Ghosal et al, 1991 ▶; Frolova et al., 1996 ▶). It is supposed that the main biological effects of Shilajit depend on the presence of fulvic acid, humic acid, and dibenzo-a-pyrones, which are carrier molecules for active components (Al-Himaidi et al., 2003 ▶). Antioxidant properties of Mumijo extract can be attributed to the presence of dibenzo-pyrones and fulvic acids (FA) (Rajesh et al., 2008 ▶).

In conclusion, the present data suggest that treatment with Mumiju prevents acetic acid-induced colitis in rats and this protective effect may be at least in part, due to its antioxidant and anti-inflammatory actions. However, further investigations are necessary to evaluate whether a similar efficacy can be achieved in other models of experimental colitis that simulate human inflammatory bowel disease and also evaluate the post-treatment effect of Mumiju in acetic acid-induced colitis.

## References

[B1] Agarwal SP, Khanna R, Karmarkar R, Anwer MK, Khar RK (2007). Shilajit: A review. Phytother Res.

[B2] Al-Himaidi AR, Mohammed U (2003). Safe use of salajeet during the pregnancy of Female mice. Online J Biol Sci.

[B3] Aiello A, Fattorusso E, Menna M, Vitalone R, Schröder HC, Müller WE (2011). Mumiju traditional medicine: Fossil deposits from antarctica (chemical composition and beneficial bioactivity). Evid Based Complement Alternat Med.

[B4] Bhattacharya SK, Sen AP, Ghosal S (1995). Effects of shilajit on biogenic free radicals. Phytother Res.

[B5] Bhattacharya SK, Ghosal S (1992). Effect of Shilajit on rat brain monoamines. Phytother Res.

[B6] Carty E, De-Brabander M, Feakins RM, Rampton DS (2000). Measurement of in vivo rectal mucosal cytokine and eicosanoid production in ulcerative colitis using ﬁlter paper. Gut.

[B7] Chavan S, Sava L, Saxena V, Pillai S, Sontakke A, Ingole D (2005). Reduced glutathione: importance of specimen collection. Indian J Clin Biochem.

[B8] Cooper HS, Murthy SN, Shah RS, Sedergran DJ (1993). Clinicopathologic study of dextran sulfate sodium experimental murine colitis. Lab Invest.

[B9] Dieleman LA, Palmen MJ, Akol H, Bloemena E, Peٌa AS, Meuwissen SG, Van Rees EP (1998). Chronic experimental colitis induced by dextran sulphate sodium (DSS) is characterized by Th1 and Th2 cytokines. Clin Exp Immunol.

[B10] Fiocchi C (1998). Inﬂammatory bowel disease: etiology and pathogenesis. Gastroenterology.

[B11] Frolova LN, Kiseleva TL (1996). Chemical composition of mumijo and methods for determining its authenticity and quality (a review). Pharm Chem J.

[B12] Garedewa A, Feist M, Schmolz E, Lamprecht I (2004). Thermal analysis of mumiyo, the legendary folk remedy from the Himalaya region. Thermochim Acta.

[B13] Ghaaazi F, Shahrokhi N, Khaksari M, Asadikaram G, Atashbar J (2018). Effect of Shilajit on the levels of pro-inflammatory and anti- inflammation cytokines in hepatic injury in male rats. Journal of Mazandaran University of Medical Sciences.

[B14] Ghannadi A, Zolfaghari B, Shamashian S (2011). Necessity, importance and applications of traditional medicine in different ethnic. J Tradit Med Islam.

[B15] Ghosal S (1990). Chemistry of shilajit, an immunomodulatory ayurvedic rasayan. Pure Appl Chem (IUPAC).

[B16] Ghosal SK, Kumar Y, Srivastava R, Goel RK, Dey R, Bhattacharya SK (1988). Anti-ulcerogenic activity of fulvic acids and 4’-methoxy-6-carbomethoxybiphenyl isolated from shilajit. Phytother Res.

[B17] Ghosal S, Lal J, Singh SK, Dasgupta G, Bhaduri J, Mukhopadhyay M (1989). Mast cell protecting effects of shilajit and its constituents. Phytother Res.

[B18] Ghosal S, Lal J, Singh SK, Goel RK, Jaiswal AK, Bhattacharya SK (1991). The need for formulation of shilajit by its isolated active constituents. Phytother Res.

[B19] Ghosal SL, Jaiswal AK, Bhattacharya SK (1993). Effects of Shilajit and its active constituents on learning and memory in rats. Phytother Res.

[B20] Goel RK, Banerjee RS, Acharya SB (1990). Antiulcerogenic and antiinflammatory studies with shilajit. J Ethnopharmacol.

[B21] Kel’ginbaev NS, Sorokina VA, Stefanidu AG, Ismailova VN (1973). Treatment of long tubular bone fractures with Mumie Assil preparations in experiments and clinical conditions. Eksp Khir Anesteziol.

[B22] Keshavarzi Z, Khaksari M, Razmi Z, Soltani Hekmat A, Naderi V, Rostami S (2012). The effects of cyclooxygenase inhibitors on the brain inflammatory response following traumatic brain injury in rats. Iran J Basic Med Sci.

[B23] Keshavarzi Z, Alikhani V, Vatanchian M, Tabatabaei Yazdi A, Bibak B, Mohebbati R (2014). Effects of Aloe Vera Gel on Gastric Acid Secretion and Colon Histopathology in Ulcerative Colitis Model induced by Acetic Acid in Rats. Zanjan University of Medical Sciences.

[B24] Loftus Jr, EV (2004). Clinical epidemiology of inﬂammatory bowel disease: incidence, prevalence, and environmental inﬂuences. Gastroenterology.

[B25] Lowry OH, Rosebrough NJ, Farr AL, Randall RJ (1951). Protein measurement with the Folin phenol reagent. J Biol Chem.

[B26] Mirza MA, Alam MN, Faiyazuddin M, Mahmood D, Bairwa R, Mustafa G (2010). Shilajit: An ancient panacea. Int J Curr Pharmaceut Rev Res.

[B27] Morris GP, Beck PL, Herridge MS, Depew WT, Szewczuk MR, Wallace JL (1989). Hapten-induced model of chronic inflammationand ulceration in the rat colon. Gastroenterology.

[B28] Nosalova V, Cerna S, Bauer V (2000). Effect of N-acetylcysteine on colitis induced by acetic acid in rats. Gen Pharmacol.

[B29] Ohkawa H, Ohishi N, Yagi K (1979). Assay for lipid peroxides in animal tissues by thiobarbituric acid reaction. Anal Biochem.

[B30] Phaechamud TJC, Wetwitayaklung P, Limmatvapirat C, Srichan T (2008). Some biological activities and safety of mineral pitch (mumiju). Silpakorn Univ Sci Technol J.

[B31] Rajesh K, Witt M, Anwer MK, Agarwal SP, Koch BP (2008). Spectroscopic characterization of fulvic acids extracted from the rock exudate shilajit. Org Geochem.

[B32] Repetto MG, Llesuy SF (2002). Antioxidant properties of natural compounds used in popular medicine for gastric ulcers. Braz J Med Biol Res.

[B33] Sartor RB (1997). Pathogenesis and immune mechanisms of chronic inﬂammatory bowel disease. The American Journal of Gastroenterology.

[B34] Shakurov AS (1965). Effect of “mumie” on bone regeneration and blood alkaline phosphatase in experimental fractures of the tubular bones. Ortop Travmatol Protez.

[B35] Shahrokhi N, Keshavarzi Z, Khaksari M (2015). Ulcer healing activity of Mumijo aqueous extract against acetic acid induced gastric ulcer in rats. J Pharm Bioallied Sci.

[B36] Spassov V (1994). Memory effects of the natural product Mumyo on the water maze in rats. Eur Neuropsychopharmacol.

